# Lay Knowledge and Beliefs Toward Dementia Among the Black African Populations in the UK and Africa: Evidence Synthesis of Qualitative Studies

**DOI:** 10.1177/21501319241291786

**Published:** 2024-10-21

**Authors:** Raphael Chinedu Mokwenye

**Affiliations:** 1 Brunel University London, Uxbridge, UK

**Keywords:** dementia, Alzheimer’s disease, lay knowledge, beliefs, Black African populations, evidence synthesis, meta-ethnography, UK, BAME

## Abstract

**Background:** The high prevalence of dementia among Black Africans, coupled with their lower engagement with dementia specialist services in the UK, underscores the urgency of understanding lay knowledge and beliefs about dementia in the group. Studies reporting lay knowledge of dementia in the UK tended to conclude that Black Africans lack dementia knowledge, presumably based on biomedical assumptions, without taking into consideration the Black Africans’ lay dementia knowledge and beliefs about dementia. The current study, therefore, investigated the Black African populations’ lay knowledge and beliefs toward dementia in Africa, comparing how this relates to the findings in the UK literature. Methodology: The researcher thoroughly searched electronic databases from September 2017 to October 2022 for qualitative research exploring how Black African populations perceived and experienced dementia, which informed the Black Africans’ dementia knowledge in Africa and the UK. The review included qualitative studies with African populations published in peer-reviewed journals with available abstracts and full text in English. Studies outside the UK and Africa, as well as quantitative research and studies with health practitioners, were excluded. A grounded theory approach informed the thematic analysis. The researcher reported the Black Africans’ lay knowledge and beliefs toward dementia, informed by participants’ experiences and meanings of dementia. Results: Twenty-two studies (n = 22) met the eligibility criteria and were appraised and included in the review. All the UK papers were a multi-modeling of ethnicity in the study. Nine studies (n = 9) were conducted in the UK. Thirteen studies (n = 13) are conducted in Africa, (5 [n = 5] studies from South Africa, 2 [n = 2] from Tanzania, 1 [n = 1] from Congo, 2 [n = 2] from Uganda, 2 [n = 2] from Nigeria, and 1 [n = 1] from Ghana). All the studies were valuable. The researcher identified and developed 4 themes as they emerged from the studies: (i) Dementia witchcraft paradigm, (ii) Dementia older age paradigm, (iii) Dementia disease and illness paradigm, and (iv) Dementia identity paradigm. Discussion/Conclusion: The lay knowledge and beliefs about dementia among Black African populations were foregrounded in sociocultural distinctiveness, and some understanding intersected with biomedical knowledge about dementia. Further empirical study in the UK is essential. Implications for policy and practice: A better understanding of lay knowledge and beliefs about dementia among Black African populations can improve dementia care, providing culturally sensitive and tailored support for Black African communities.

## Background

For research to effectively inform knowledge of how different people understand and experience dementia, one would need to understand the ethnicity and cultural backgrounds of the populations.^1,2^ For example, in Africa, knowledge about dementia can be informed by sociocultural factors such as occupation, education, gender roles, age, spirituality and religion,^3^ and cultural contexts that are socially transmitted from one generation to another^4,5^ These sociocultural contexts, such as witchcraft^6^ or spirituality and religion impacted African people’s understandings, meanings, and beliefs about dementia,^7-10^ drawing upon values, customs, relationships, and practices that shape the way Africans communicate, think, and behave that characterize Black Africans, particularly with people with dementia and caregiving for dementia.^11-13^

Thus, this current work is designed to understand and improve the health of BAME (defined as all ethnic groups except White ethnic groups) communities in the UK focused on dementia and the Black African populations to inform dementia practice and policy. Equal opportunity to access dementia services is a priority and essential for a racially and ethnically diverse population in the UK, including those conducting dementia research.^14^ Dementia services in the UK require an understanding of lay dementia knowledge to provide patients, particularly Black African communities, with the dementia services needed.^15^

Studies in the UK suggested that Black Africans lack dementia knowledge^16^ and that African populations rely on religious support and are underrepresented in dementia specialist services.^17^ When Black Africans in the UK use dementia specialist services, they usually do so later in the condition^18,19^ and are often at higher risk of not being diagnosed and prescribed the appropriate care and medication for dementia.^19^

In the UK, about 25 000 BAME (Black, Asian and Minority Ethnic) population are living with dementia, and this has been projected to increase to about 172 000 by 2050.^20,21^ The African population represents a significant population in the UK. According to the 2021 Census, approximately 2.4 million Black individuals reside in the UK, which accounts for 4.2% of the population. Africans comprise more than half of this population, with 1.5 million people representing 2.5%, increasing from 1.8% in 2011. Black African people are the largest ethnic group within this population and are comprised mostly of Nigerians (0.5%), Somalis (0.3%), and Ghanaians (0.2%).^22^ Thus, Black Africans can lead to increased dementia prevalence in the BAME in the UK.^23,24^

Moreover, average life expectancy has improved globally.^25^ It has increased in Black African populations and was expected to rise above the average of 65 years in the UK due to improved quality of life and access to essential health services.^26^ Therefore, we would expect dementia to rise among the Black African populations in the UK, underscoring the urgency of this current research to explore the lay knowledge and beliefs toward dementia among Black Africans informing dementia practice and policy.

This study systematically reviews the literature on lay knowledge and beliefs about dementia using evidence synthesis methodology.^27,28^ The research identifies the qualitative studies relevant in Africa and the UK and identifies gaps in the UK studies on dementia among the African population. This review excluded studies outside of the UK and Africa because the research is focused on the UK to inform the UK dementia services. Even though most of the qualitative studies on dementia are conducted in Africa, the UK, and the United States. The evidence provided in most studies in the United States was on Black Americans.^14^ The data did not apply to the UK’s Black African context, which is the population of interest in this study. Therefore, research from the United States was excluded from the review to enable the current researcher to obtain the best evidence and reach the best conclusion. Nonetheless, the researcher ensured that the review process was robust. Further, it emphasizes the fact that context is essential in this study.

## Research Context

Lay people from Black African communities may lack biomedical knowledge of dementia. However, Black Africans have lay knowledge and beliefs about dementia based on a shared sociocultural understanding of the dementia phenomenon in the Black African communities that frame or shape their dementia service use and non-use pattern within a broader sociocultural context.^29,30^ So, our working definition of lay knowledge and beliefs about the definition is the “general cultural stock of knowledge”^31^ held by the local members of the Black African communities who are not medical or health practitioners. Of course, everyone has access to their own type of lay knowledge.^32^

## Review Question

The research used evidence synthesis to explore lay knowledge and beliefs about dementia among Black African populations in the UK and Africa. It aimed to identify gaps in UK studies and understand how dementia is constituted and constructed among Black Africans.

### Review Question

What are the lay knowledge and beliefs toward dementia among the Black African populations in the UK and Africa?

### Aim

The review aims to identify gaps in the UK literature.

### Objective

The objective is to understand dementia services for the Black African populations in the UK by understanding how dementia is constituted and constructed among the Black African populations in the UK and African studies.

## Methods

This study systematically reviews the qualitative studies on lay knowledge and beliefs about dementia using an evidence synthesis principle^27,28^ to address the research question.

### Search Strategy

The research strategy covers 2 areas of literature search. Firstly, a comprehensive literature search on the research topic was performed using Google Scholar to overview the research topic. Google Scholar is a part of the popular WWW search engine, which means there were no limits on the languages covered, keywords allowed per search, and the list of covered journals.^33^ Given the vast amount of literature, most papers were outside this study’s scope, and it was impossible to review them systematically. Nevertheless, most of the studies on dementia in the African population were conducted in Africa, the UK, and America. However, the researcher excluded studies conducted in the United States on the grounds of transferability since the focus was on Black Africans and the UK. Dementia studies in the United States were mainly conducted on African Americans^14^ and not within the context of Black Africans.

A systematic literature search was conducted in the second search to identify relevant studies.^27,28^ Four databases were used to find peer-reviewed articles and provide literature on the topic. The databases were selected to enable a search strategy for research published in a broad disciplinary tradition. The search was conducted from 3 September 2017 to 17 October 2022. Five years of searching databases and reporting ensured the robustness of the review process. This added rigor and quality to the study and helped the researcher achieve trustworthiness. It also helped the researcher learn and appreciate the breadth and depth of dementia knowledge and access current studies on the topic. The researcher used search words as identified inductively from the literature, and were “dementia,” “Alzheimer’s,” “culture,” “religion,” “anthropology,” “belief,” “perception,” “knowledge,” “lay,” “view,” “stigma,” “attitude,” “constructionism,” “Africa,” and “UK.” Each keyword and the phrase were initially used singly and combined using the Boolean operators “AND” and “OR.” Thus, the researcher undertook the search and launched the literature quality appraisal. A set of the combined search terms is as indicated and was used in each database.

(Dementia OR Alzheimer*) AND (Cultur* OR Relig* OR Anthropol*) AND (Belief* OR Perception* OR Knowledge OR Lay OR View* OR Attitude* OR Stigma OR Constructionism) AND (Africa OR UK).

The researcher searched 4 databases: Scopus, Web of Science, Medline, and PubMed. Scopus was used because of the interdisciplinary nature of the study topic. Scopus delivers the world’s research output in medicine and social sciences. It covers life sciences, social science, and health science and covers a broader journal range in keyword searching and citation analysis.^33^ Web of Science was used because the database supports various scientific tasks across diverse knowledge domains. It covers the oldest publications, indexes, and archived records dating to 1900.^33^ Hence, the researcher used PubMed and Medline because of the study topic’s relation to clinical medicine and health science. PubMed focuses on medicine and biomedical sciences,^33^ although PubMed has an interface that searches Medline.

The 4 databases were selected to enable a search strategy for research published in various disciplinary traditions, including broader social sciences, clinical medicine, and health sciences. These combined characteristics of databases enhanced the utility of medical literature and social sciences in meeting this study’s academic and professional needs. In each database, date limits were not applied to the search to consider a broader perspective. The set of papers on return was screened for eligibility to be included in the review. Nonetheless, this study literarily used only 3 electronic databases (see Table 1) because PubMed was an interface that searched Medline. So, Medline is the same as PubMed. The researcher used Google Scholar to overview the research topic. The review augmented the electronic search with backward and forward reference searching of included articles and carried out grey and manual book searches.

**Table 1.** Search Strategy and Results.

Databases	Keyword search	Search with (last searched on 17 October 2022)	Operators/boolean/phrase	Total number of hits
Scopus	Dementia, Alzheimer’s, Culture, Religion, Anthropology, Belief, Perception, Knowledge, Lay, View, Stigma, Attitude, constructionism, Africa, UK	Article title, Abstract, Keywords	(Dementia OR Alzheimer*) AND (Cultur* OR Relig* OR Anthropol*) AND (Belief* OR Perception* OR Knowledge OR Lay OR View* OR Attitude* OR Stigma OR constructionism) AND (Africa or UK).	120
Web of Science	Dementia, Alzheimer’s, Culture, Religion, Anthropology, Belief, Perception, Knowledge, Lay View, Stigma, Attitude, constructionism, Africa, UK	Topic	(Dementia OR Alzheimer*) AND (Cultur* OR Relig* OR Anthropol*) AND (Belief* OR Perception* OR Knowledge OR View* OR Attitude* OR Stigma OR constructionism) AND (Africa OR UK).	79
PubMed/Medline	Dementia, Alzheimer’s, Culture, Religion, Anthropology, Belief, Perception, Knowledge, Lay View, Stigma, Attitude, constructionism, Africa, UK	All field	(Dementia OR Alzheimer*) AND (Cultur* OR Relig* OR Anthropol*) AND (Belief* OR Perception* OR Knowledge OR View* OR Attitude* OR Stigma, constructionism) AND (Africa or UK).	284
Total databases searched = 3				Total hits = 483

### Eligibility Criteria

To be included in the review, papers needed to meet the eligibility criteria for inclusion^27,28^ and papers that did not meet the eligibility criteria were excluded from the research (see Table 2). The papers included in the review were published peer-reviewed articles with available abstracts and full text written in English. The review excluded studies outside the UK and Africa. The focus of this study is to identify gaps in the UK. As noted, some of the few studies on African populations were conducted in the United States among African Americans rather than Black Africans.^14^ Therefore, this study excluded papers conducted in the United States on the grounds of transferability and confirmability.^34^ The evidence provided in the studies conducted in the United States does apply to the UK context using the trustworthiness discourse.^34-36^

**Table 2.** Eligibility Criteria for Inclusion and Exclusion Using the APSO Framework.

Inclusion criteria	Exclusion criteria
Articles (**A**): • Peer-reviewed articles • Published journal articles (no date limit) • Available abstract • Available full-text • Papers written in English Population (**P**): • Studies conducted in Africa with African populations. • Studies conducted in the UK with African populations. Study design (**S**): • Primary qualitative studies • Primary mixed-methods studies Outcome (**O**): • Dementia services	Articles (**A**): • Non-peer-reviewed articles • Nonavailable abstract • Nonavailable full-text • Papers not written in English • Letters • Commentaries • Conference proceedings • Media articles Population (**P**): • Studies with African populations conducted outside Africa. • Studies with African populations conducted outside the UK. • Studies with health practitioners. Study design (**S**): • Quantitative studies • Systematic reviews • Traditional reviews Outcome (**O**): • Not on dementia services

Furthermore, this study included only research reporting qualitative studies as this was the methodology appropriate to the review question.^37^ Therefore, quantitative studies are excluded. However, quantitative analysis can consist of expressions of attitude and epidemiology, and often close-ended and expressed numerically to ensure statistical representation^38^ rather than decisions gathered through contextualized inquiries or interviews to learn about feelings, meanings, attitudes, behaviors, and experiences^39-41^ of dementia. Therefore, the review included mixed-method studies^38^ of dementia because when mixed studies are used, the data can gather the target audience’s demographic experiences, attitudes, feelings, behaviors, and meanings of dementia.^42^

In addition, the inclusion and exclusion criteria are used when screening results from the initial database to ensure relevance to the review question. The researcher utilized the APSO checklist, which he developed inductively from the papers, and further, the study used the CASP qualitative checklist.^43^ The tools were an initial practical framework to appraise the evidence and ensure relevance to answering the review question. Finally, the researcher uses the trustworthiness criteria (EOR) to evaluate the qualitative studies.^34,44^ The APSO checklist is as demonstrated.

## APSO Checklist

The APSO checklist is developed inductively by considering the papers and is like the PICO strategy.^45,46^ The researcher wanted to ensure that the studies considered for this review contained the right populations (see Table 2). This review is not about dementia intervention but about dementia knowledge. Indeed, many UK papers were on BAME. However, only a few had Black Africans as respondents or participants, and the researcher wanted to ensure the study’s outcome was based on dementia services. Using the APSO checklist ensures the appropriateness of the articles, population, study design, and outcome of the research and offers rigor and the ability to synthesize the evidence needed for the review using this criterion as highlighted:

**A** = Article **P** = Population **S** = Study design **O** = Outcome
**A** (Articles)
 • Is it a published journal article?
 • Is the abstract available?
 • Is the full text available?
 • Is the article written in English?
**P** (Population)
 • Is the study conducted in Africa with African populations?
 • Is the study conducted in the UK with African populations?
**S** (Study design)
 • Is it a qualitative study?
 • Is it a quantitative study?
 • Is it a mixed-methods study?
**O** (Outcome)
 • What is the outcome of the study?
 • Is the outcome relevant to the current study?

### The Identification of Studies to be Included or Excluded

The study identification process is divided into 2-stage operations:

(i) Level 1 screening, and
(ii) Level 2 screening process.

Level 1 was done using the publication modality, whilst the level 2 process was done using the APSO checklist as illuminated above, and then the CASP^43^ qualitative checklist for quality appraisal of the selected articles.

In the level 1 screening, the papers identified from the 3 central databases were assessed using the APSO checklist, for example, based on peer-reviewed journals, available abstracts, available full text, and establishing that the papers were written in English (as illustrated in Tables 2 and 3).

**Table 3.** Screening of the Included Papers Using the APSO Checklist.

		Articles				Population	Study design	Outcome
		Journal	Abstract	Full text	English			
1	Adamson (2001)	Yes	Yes	Yes	Yes	African Caribbean	Qualitative	Dementia services
2	Adamson and Donovan (2005)	Yes	Yes	Yes	Yes	African Caribbean	Qualitative	Dementia services
3	Adebiyi et al (2016)	Yes	Yes	Yes	Yes	Africans	Mixed	Dementia services
4	Agyeman et al (2019)	Yes	Yes	Yes	Yes	Africans	Qualitative	Dementia services
5	Armstrong et al (2022)	Yes	Yes	Yes	Yes	Africans Caribbean	Qualitative	Dementia services
6	Baghirathan et al (2020)	Yes	Yes	Yes	Yes	African Caribbean	Qualitative	Dementia services
7	Berwald et al (2016)	Yes	Yes	Yes	Yes	Black Africans	Qualitative	Dementia services
8	Botsford et al (2011)	Yes	Yes	Yes	Yes	African Caribbean	Qualitative	Dementia services
9	Gurayah (2015)	Yes	Yes	Yes	Yes	Africans	Qualitative	Dementia services
10	Hindley et al (2016)	Yes	Yes	Yes	Yes	Africans	Qualitative	Dementia services
11	Jacobs et al (2022)	Yes	Yes	Yes	Yes	Africans	Qualitative	Dementia services
12	Kakongi et al (2020)	Yes	Yes	Yes	Yes	Africans	Qualitative	Dementia services
13	Kehoua et al (2019)	Yes	Yes	Yes	Yes	Africans	Qualitative	Dementia services
14	Lawrence et al (2008)	Yes	Yes	Yes	Yes	African Caribbean	Qualitative	Dementia services
15	Mahomed and Pretorius (2022)	Yes	Yes	Yes	Yes	African	Qualitative	Dementia services
16	Mahomed and Pretorius (2021)	Yes	Yes	Yes	Yes	Africans	Qualitative	Dementia services
17	Mkhonto and Hanssen (2018)	Yes	Yes	Yes	Yes	Africans	Qualitative	Dementia services
18	Mukadam et al (2011)	Yes	Yes	Yes	Yes	Africans or Caribbean	Qualitative	Dementia services
19	Mushi et al (2014)	Yes	Yes	Yes	Yes	Africans	Qualitative	Dementia services
20	Nwakasi et al (2021)	Yes	Yes	Yes	Yes	Africans	Qualitative	Dementia services
21	Owokuhaisa et al (2020)	Yes	Yes	Yes	Yes	Africans	Qualitative	Dementia services
22	Parveen et al (2017)	Yes	Yes	Yes	Yes	Africans and Caribbean	Mixed	Dementia services

In the level 2 screening, the researcher removed the duplicates, and the remaining papers were screened based on relevance to the study using the APSO checklist (please refer to Table 3). The use of the APSO checklist by the researcher was essential because the evidence synthesis needs to be directly relevant to the population of study and phrased in such a way as to facilitate answering the research question. Therefore, the APSO checklist made the literature review process more transparent and provided quality and rigor in the review process. It focused the evidence synthesis on peer-reviewed articles with African people as respondents or participants in the qualitative studies and excluded studies outside of the UK and Africa. More so, the researcher was interested in the outcome of the dementia services. The researcher’s professional and personal experiences of providing care for a family member with dementia, his clinical background, and being a Black African in the UK situated his positionality^47,48^ and biases in the review and met his interest as an insider and outsider in the review and evaluation process.^49,50^

So, the inclusion and exclusion criteria were used when screening results from the initial database. In addition to the search and ensuring relevance to the research question, the researcher used the APSO checklist (see Table 3) when reading titles and abstracts and the full article to ensure relevance and the ability to answer the research question. The included articles were then appraised using the qualitative checklist of the Critical Appraisal Skills Programme^43^ (see Table 4). The researcher used these checklists to screen and appraise the papers methodologically and evaluate the quality of the included articles.

**Table 4.** Quality Assessment of Included Studies Using the Critical Appraisal Skills Programme: Qualitative Checklist.

Papers	Quality control											Quality outcome
	1	2	3	4	5	6	7	8	9	10	Total	
Adamson (2001)	1	1	1	0.5	0.5	0	0	0.5	0.5	1	6	Moderate
Adamson and Donovan (2005)	1	1	1	0.5	0.5	0	0	0.5	0.5	1	6	Moderate
Adebiyi et al (2016)	1	1	1	1	1	0	1	1	1	1	9	High
Agyeman et al (2019)	1	1	1	1	1	0	1	1	1	1	9	High
Armstrong et al (2022)	1	1	1	0.5	0.5	0	1	0.5	0.5	1	7	High
Baghirathan et al (2020)	1	1	1	0.5	0.5	1	1	0.5	0.5	1	8	High
Berwald et al (2016)	1	1	1	1	1	0	1	0.5	0.5	1	8	High
Botsford et al (2011)	1	1	1	0.5	0.5	0	1	0.5	0.5	1	7	High
Gurayah (2015)	1	1	1	0	0	0	1	0	0	1	5	Moderate
Hindley et al (2016)	1	1	1	1	1	0	1	1	1	1	9	High
Jacobs et al (2022)	1	1	1	1	1	0	1	1	1	1	9	High
Kakongi et al (2020)	1	1	1	1	1	0	1	1	1	1	9	High
Kehoua et al (2019)	1	1	1	0	1	0	1	1	1	1	8	High
Lawrence et al (2008)	1	1	1	0.5	0.5	0	0	0.5	0.5	1	6	Moderate
Mahomed and Pretorius (2022)	1	1	1	1	1	0	1	1	1	1	9	High
Mahomed and Pretorius (2021)	1	1	1	0.5	0.5	0	1	0.5	1	1	7.5	High
Mkhonto and Hanssen (2018)	1	1	1	0.5	0.5	0	1	0.5	1	1	7.5	High
Mukadam et al (2011)	1	1	1	0.5	0.5	0	1	0.5	0.5	1	7	High
Mushi et al (2014)	1	1	1	1	1	0	1	1	1	1	9	High
Nwakasi et al (2021)	1	1	1	1	1	0	1	1	1	1	9	High
Owokuhaisa et al (2020)	1	1	1	1	1	0	1	1	1	1	9	High
Parveen et al (2017)	1	1	1	0	0	0	1	0.5	0.5	1	6	Moderate

Indeed, 22 papers were included in the review (n = 22), although Armstrong et al’s^51^ study and Mkhonto and Hassen’s^8^ paper were problematic. Armstrong et al’s^51^ study explored the perspectives of Black and Asian people living with dementia and their carers in the UK. Of the 15 participants, 7 were of Black ethnicity, and all were Caribbean. Some participants reported birth in African countries such as Kenya and Uganda, but all those who reported birth from the African countries defined themselves as South Asians. Nevertheless, the researcher included the UK study in the review because the paper contains Black Caribbean respondents, and the findings were relevant to the current study. Whilst Mkhonto and Hassen’s^8^ paper involved 19 nurses; even though this current review excluded studies with a health professional, the researcher included the paper because the study involved 18 family members; 2 of the 18 family members were of Black African background and were not health professionals.

### Quality Appraisal

The included studies were assessed against the Critical Appraisal Skills Programme.^43^ The CASP systematic review qualitative checklist highlighted ten questions to help make sense of the review papers as a quality measure. The appropriateness of the methodology, for example, research design, research methods, and findings were considered.^43^ For instance, 3 broad issues were considered when appraising the included studies:

(i) Were the results of the study valid?
(ii) What are the findings?
(iii) Will the findings help understand dementia phenomena?

Subsequently, the articles were judged as high, moderate, or low quality. No low-quality papers were identified in the studies (see Table 4).

### CASP (2018) Checklist

1. Was there a clear statement of the aims of the research?
2. Is a qualitative methodology appropriate?
3. Was the research design appropriate to address the aims of the research?
4. Was the recruitment strategy appropriate to the aims of the research?
5. Was the data collected in a way that addressed the research issue?
6. Has the relationship between the researcher and participants been adequately considered?
7. Has the ethical issue been taken into consideration?
8. Was the data analysis sufficiently rigorous?
9. Is there a clear statement of findings?
10. How valuable is the research?

### Quality Assessment

The CASP checklist is designed to be used as a scoring system. However, the researcher adapted the scores by assigning a maximum of 1 point per item,^14^ with a possible score ranging from 0 to 10 (lowest to highest quality, respectively). He gave a partial point (0.5) if the study population was not entirely Africans but a multi-modeling of ethnicity and if the data analysis failed to provide separate findings for the African people in the study. Therefore, a score of 7 to 10 was judged as a high-quality study, 5 to 6 was evaluated as a moderate-quality study, and a score below 5 was considered a low-quality study. Still, no low-quality paper was identified in the study. Seventeen papers (n = 17) were regarded as high quality, and 5 (n = 5) papers were considered moderate-quality studies. Baghirathan et al’s^52^ paper was the only study that assessed the relationship between participants and the researcher’s biases. Therefore, all the papers needed more positionality and reflexivity. Concerning the Armstrong et al’s^51^ and Mkhonto and Hassen’s^8^ studies, although the papers were problematic, the studies were included, and their findings were significant in answering the research questions.

### Evaluation Discourse (EOR)

The studies were evaluated further by drawing on philosophical and evaluation discourses,^53-55^ based on the research, epistemological and ontological concepts,^56^ and mainly used trustworthiness criteria.^34,44^ The criterion has been generally accepted as the platform for evaluating qualitative studies. It offered sufficient flexibility to accommodate the best evidence and practices for achieving rigor and trustworthiness within the qualitative paradigm. It advocates for 5 key concepts:

1. Credibility
2. Transferability
3. Dependability
4. Confirmability
5. Authenticity

These 5 key concepts are used to evaluate the included literature, assess whether the studies were guided by theory, methods, and empirical data, and assess their outcomes and relevance.

Overall, twenty-two studies (n = 22) met the eligibility criteria and were appraised and included in the review (Table 4). All the UK papers were a multi-modeling of ethnicity in the study. Nine studies (n = 9)^16-18,51,52,57-60^ are research conducted in the UK. Thirteen studies (n = 13)^7-13,61-66^ are research conducted in Africa (5 [n = 5] studies from South Africa, 2 [n = 2] studies from Tanzania, 1 [n = 1] study from Congo, 2 [n = 2] studies from Uganda, 2 [n = 2] studies from Nigeria, and 1 [n = 1] study from Ghana].

All the studies were valuable based on the topic, research design and methods, and the outcome of the studies, offering essential insights into dementia services in Africa and the UK with African populations. However, they were limited in methodology and lacked the researcher’s positionality and biases (see Table 5). All the UK papers were multi-ethnic studies, and the studies did not separate the findings of the Africans from the Caribbean people. Therefore, based on the discourse criterion, the findings were valuable but unreliable. Nevertheless, Berwald et al^16^ suggested that Black Africans lacked dementia knowledge. This finding is invalid as the study had no separate data for the Black African population. Thus, the conclusion drawn from the study is not credible and lacks authenticity for the Black African community in the UK (Table 6).

**Table 5.** Identified Gaps in the Literature and Plans for an Empirical Study in the UK.

	Studies	Identified gaps	Plans for empirical study	Study design	Methods	Sampling	Sample size	Plans for analysis
1	All the UK studies	All the studies were multi-modeling of ethnicity, and the results did not separate Black Africans from the Caribbean. Thus, the studies lack trustworthiness criteria (Lincoln and Guba, 1985).	To explore dementia among Black African populations in the UK	Qualitative	Semi-structured interviews	Purposive	15-56	A grounded theory approach will be used to inform the thematic data analysis
2	All the UK studies	The studies did not explore lay knowledge and beliefs about dementia among Black Africans in the UK.	To explore lay knowledge and beliefs toward dementia among Black African populations in the UK	Qualitative	Semi-structured interviews	Purposive	15-56	A grounded theory approach will be used to inform the thematic data analysis
3	All the UK studies	The studies lacked theory, methods, and empirical evidence, which made them unclear and invalid for Black Africans in the UK (Lather, 1986).	To develop a conceptual framework for the study and to collect empirical data	Qualitative	Semi-structured interviews	Purposive	15-56	A grounded theory approach will be used to inform the thematic data analysis
4	Armstrong et al (2022).	All the participants who reported birth from African countries defined themselves as South Asian.	To explore lay knowledge and beliefs toward dementia among Black African populations in the UK	Qualitative	Semi-structured interviews	Purposive	15-56	A grounded theory approach will be used to inform the thematic data analysis
5	All the African and UK studies	The studies lacked the researcher’s positionality (epistemological and ontological stance).	To highlight the current researcher’s positionality.	Qualitative	Semi-structured interviews	Purposive	15-56	A grounded theory approach will be used to inform the thematic data analysis.

**Table 6.** Extracted Data From the Included Papers.

	Study	Aim	Analysis	Sample size	Black participants	Ethnicity involved	Country
1	Adamson (2001)	Explored awareness, recognition, and understanding of dementia	GT	30 15F 3M	African Caribbean	BAME	UK
2	Adamson and Donovan (2005)	Explored the experiences of dementia	GT	36	African Caribbean	BAME	UK
3	Adebiyi et al (2016)	Explored enacted and implied stigma for dementia	Thematic analysis	48	Africans	Africans	Nigeria
4	Agyeman et al (2019)	Explored sociocultural beliefs, perceptions, and behavior toward dementia	Thematic analysis	28	Africans	Africans	Ghana
5	Armstrong et al (2022)	Explored experiences of dementia	Thematic analysis	15	Africans Caribbean	BAME	UK
6	Baghirathan et al (2020)	Explored the experiences of dementia	GT	103	African Caribbean	BAME	UK
7	Berwald et al (2016)	Explored the barriers to help-seeking for memory loss/dementia	Thematic analysis	50 30F 20M	Black Africans	BAME	UK
8	Botsford et al (2011)	Explored the experience of dementia	CGT	43	African Caribbean	BAME	UK
9	Gurayah (2015)	Explored the experience of dementia	Thematic analysis	5 4F 1M	Africans	Africans	South Africa
10	Hindley et al (2016)	Explored knowledge, beliefs, and treatment toward dementia	Thematic analysis	56	Africans	Africans	Tanzania
11	Jacobs et al (2022)	Explored knowledge, attitudes, and beliefs toward dementia	Thematic analysis	52	Africans	Africans	South Africa
12	Kakongi et al (2020)	Explored the understanding of care-seeking for Alzheimer’s disease and dementia	Thematic analysis	30	Africans	Africans	Uganda
13	Kehoua et al (2019)	Explored the perceptions and social representations of dementia	Thematic analysis	93	Africans	Africans	Republic of Congo
14	Lawrence et al (2008)	Explored attitudes and experiences toward dementia	GT	32 25F 7M	African Caribbean	BAME	UK
15	Mahomed and Pretorius (2022)	Explored awareness and behavior toward Alzheimer’s disease	Thematic analysis	11	Africans	Africans	South Africa
16	Mahomed and Pretorius (2021)	Explored experiences of dementia	Thematic analysis	30	Africans	Africans	South Africa
17	Mkhonto and Hanssen (2018)	Explored knowledge and beliefs toward dementia	Thematic analysis	37	Africans	Africans	South Africa
18	Mukadam et al (2011)	Explored attitudes and behavior toward dementia	Thematic analysis	18 13F 5M	Africans or Caribbean	BAME	UK
19	Mushi et al (2014)	Explore perceptions and representations of dementia	Content analysis	41 26F 15M	Africans	Africans	Tanzania
20	Nwakasi et al (2021)	Explored attitudes toward dementia	Thematic	12	Africans	Africans	Nigeria
21	Owokuhaisa et al (2020)	Explored perceptions of dementia	Thematic analysis	22 8F 14M	Africans	Africans	Uganda
22	Parveen et al (2017)	Explored perceptions of dementia	Thematic analysis	175	Africans Caribbean	BAME	UK

Furthermore, the studies show that the African populations used religious and spiritual support from churches and relied on friends and family for dementia advice, support and care.^16,17^ This suggests that the respondents have knowledge and beliefs about dementia that frame or shape their dementia service use and non-use within a broader sociocultural context. So, the current researcher considers that the participants have lay knowledge and beliefs about dementia, which the authors did not consider. Indeed, dementia can be transcendental,^67^ and the lay concepts of dementia are holistic.^5,29,30^

However, the data illuminated a perceived lack of cultural sensitivity from GPs, and the respondents perceived that GPs lacked dementia knowledge.^17^ This suggests epistemic tensions between biomedical and lay knowledge^68^ about dementia in the Black African community and that medical doctors and laypeople could view dementia from different frames of reference and thus provide the reason why participants may have had negative interactions with their GPs.^17^ Indeed, Black Africans in London presented late to the inner London memory services.^19^ Nonetheless, the current researcher is a medical doctor and an applied medical anthropologist, which defines his positionality and biases in this study, and he believes the biomedical and lay concepts can interact as delineated by the concept of intersectionality.^69^ Let us note that researchers’ backgrounds, positionality, and biases can impact their research approach and outcome and determine whose voices are heard.^70^

Furthermore, the included literature was not confirmable in the study with demographic data across the entire research. Most studies lacked demographics such as occupation, gender, age, religion, education, and socioeconomic status. We expect the knowledge of dementia to vary with demographics, and nonconformity may be rare. Although, some studies (eg, Nwakasi et al,^10^ Jacobs et al,^13^ Mukadam et al,^18^ Armstrong et al,^51^ Kehoua et al,^63^ Kakongi et al,^64^ Mahomed and Pretorius,^65,66^) provided some vital demographic data. However, it was difficult to draw a separate conclusion for Black African populations in the study. Of course, in the UK, one would expect dementia knowledge among Black African populations to vary depending on educational level, occupation, age, gender, and immigration status.

In addition, 5 UK studies (n = 5) were conducted more than 10 years ago,^18,57-60^ suggesting that the data is undependable and may lack credibility for the current Black African populations in the UK because dementia knowledge may have evolved.^17^ This suggests an essential need for further research on the topic. It appears as if dementia knowledge is embedded in the respondents’ culture, and the Black Africans in the UK may have languages, narratives or metaphors for dementia that need further exploration.

**Figure 1.**
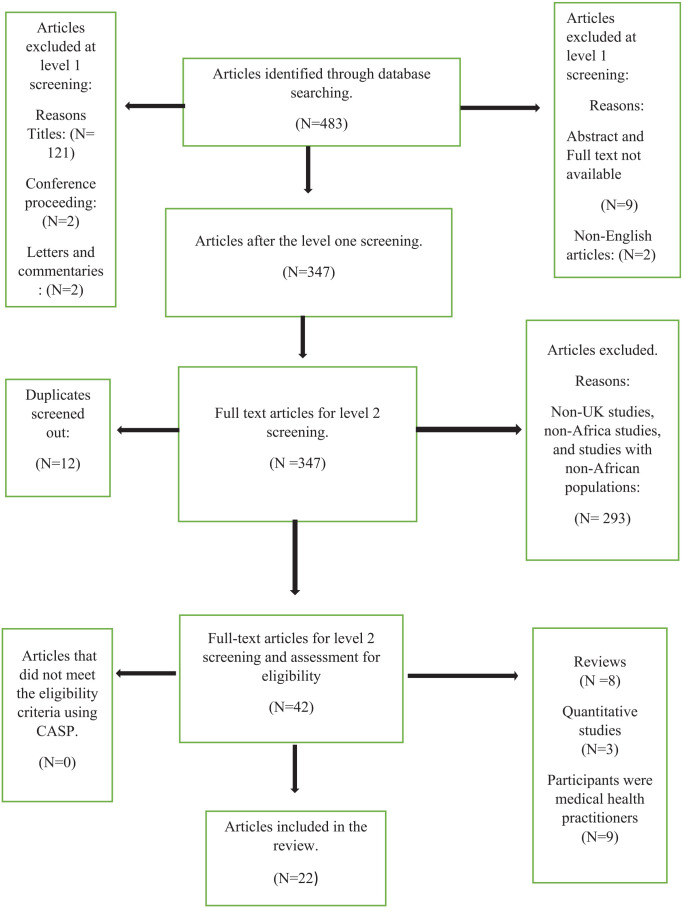
PRISMA diagram of the selection of studies.

Finally, the total number of respondents (n = 222) in all the UK studies added together was 222, with only 38 participants (n = 38) fluidly defined as Africans, constituting just about 17% of the total respondents in the UK study. However, all the African studies were of African background, comprising 465 Black African participants (n = 465) for the qualitative data. Therefore, 17% is a meager percentage, so this study considers the UK data not robust and lacks credibility for Black African populations. Thus, the UK studies’ findings are undependable and do not wholly inform us about the Black African population’s lay knowledge and beliefs toward dementia. Indeed, the UK literature lacks confirmability and authenticity for the Black African communities in the UK (Figure 1).

## Thematic Analysis

Data analysis was guided by Braun and Clarke’s^39^ recommendations on synthesizing qualitative research using thematic analysis. The researcher used the reflexive thematic analysis,^39-41,71,72^ informed by a grounded theory approach.^73,74^ The analysis is conducted to answer the review question thematically. The procedure was drawn upon in the thematic coding and analysis of the included studies and in identifying the themes and patterns or meanings across the data.^41,72,73^ The researcher analyzed vital empirical data that was accessible in the included papers. The analysis was driven by the participants’ own words rather than the researcher’s interpretation of the data.^41^ Hence, the researcher included direct quotations from the UK and African studies in his analysis and coded the extracted data line by line and between as necessary to answer the review question. Braun and Clarke’s thematic analysis method is an iterative and reflexive process consisting of 6 steps: (i) Familiarization, (ii) Coding, (iii) Generating themes, (iv) Reviewing themes, (v) Defining themes, (vi) Writing up.

The method was further enhanced by a similar process of identifying theories through analyzing the data,^74,75^ which involved familiarizing with the data, focus coding, theoretical coding, categorizing, and thematic coding through inductive reasoning.^74^ Indeed, as the researcher coded the included studies’ empirical data, embedded concepts became apparent and emerged from the data. Then, the researcher deductively assigned theories to the data.^76^

This grounded approach provided a means of constructing methods^77,78^ to better understand how dementia is constituted and constructed by Black Africans in the included studies. The process enabled the researcher to understand the socially shared constructions that underline the individual’s or group’s behavior and the dementia reality of the participants across the papers. The researcher coded the participants’ words as evidence using colors and managed the data using Excel.

## Step-by-Step Guide to the Thematic Analysis Informed by Grounded Theory Approach

Through an iterative and reflexive process of comparing the empirical data from the studies^39-41,71,72^ informed by the grounded theory approach,^74,75^ the researcher produced codes inductively, identified and refined the themes based on the empirical evidence as demonstrated in the steps:

### Step A: Open Coding

The researcher reviewed all the included studies to identify emerging codes, patterns, and themes. The process involved “familiarisation” and “constant comparative” analytical processes, which allowed the reviewer to move back and forth constantly across the studies and ensure that the complexity of the data was represented in the analysis. The step was fast and loose.

### Step B: Theoretical Coding

The researcher then developed codes that went beyond the explicit content of the data by comparing findings across the studies. The latent codes invoke a conceptual/theoretical framework identifying the assumptions underpinning the data.

### Step C: Focus Coding

This step allowed the reviewer to perform a second coding round, providing an interpretative lens to the participants’ own words or excerpts. The process involved finding patterns, renaming the codes, continuously comparing data, and refining and getting codes closer to forming a category or a new theme.

### Step D: Category Coding

The researcher turned the focus code into a category/theme by charting and grouping similar codes.

### Step E: Thematic Coding

This is the step in which the reviewer defines the themes and patterns across the dataset (unifying/mapping codes) and ensures that the excerpts point to the same underlying idea or meaning, with the intent of a continuous analysis within the structure.

### Step F: Managing the Data and Writing the Report

Excel was used to manage the data and facilitated the data analysis, as illuminated in the audit trail:

1. **Participants’ own words as evidence:** *You know in the village, they will think that somebody is bewitching [or casting evil spell on] that person [with dementia]* (10 p. 1452).
**• Open coding**: Bewitching
**• Theoretical coding:** Cultural construct
**• Focus coding:** Dementia is witchcraft
**• Category:** Spirit
**• Theme:** Witchcraft paradigm
**2. Participants’ own words as evidence:** *We call it old age, when a person becomes elderly, his knowledge reduces and he starts forgetting*^9^ (p. 3).
**• Open coding:** Old age
**• Theoretical coding:** Lay concept
**• Focus coding:** Dementia is old age
**• Category:** Body
**• Theme:** Older age paradigm
**3. Participants’ own words as evidence:** *Another thing which causes forgetfulness among the elderly, there is when you find a person has suffered from a disease for a long time like cancer and he takes different types of medicine*^9^ (p. 4).
**• Open coding:** Disease
**• Theoretical coding:** Biomedical concept
**• Focus coding:** Dementia is a disease
**• Category:** Mind
**• Theme:** Disease and Illness paradigm
**4. Participants’ own words as evidence:** *And if you see her, you won’t even know that she is having such sickness*^10^ (p. 1453).
**• Open coding: Sickness**
**• Theoretical coding:** Identity theory
**• Focus coding:** Dementia is a sickness
**• Category:** Body
**• Theme:** Identity paradigm

The empirical data presented in the literature is a subsample of the whole dataset used in the included articles (see Table 7).

**Table 7.** Thematic Analysis (Codes and Themes).

Participants’ own words as evidence	Open coding	Theoretical coding	Focus coding	Category	Themes
“. . . bewitching [or causing evil spell on] that person [with dementia]” (Nwakasi et al, 2021, p. 1452)^11^	Bewitching	Cultural construct	Dementia is witchcraft	Spirit	Witchcraft paradigm
“. . . [sorcerer/witch] is trying to manipulate her [person with dementia].” (Nwakasi et al, 2021, p. 1453)^11^	Sorcerer/witch	Cultural construct	Dementia is witchcraft	Spirit	Witchcraft paradigm
“They say ‘that one has been bewitched . . .” (Mkhonto and Hassen, 2018, p. 172)^8^	Bewitched	Cultural construct	Dementia is witchcraft	Spirit	Witchcraft paradigm
“. . . no definition of dementia, an old person is starting to forget, [it’s] part of growing old” (Berwald et al, 2016, p. 11)^16^	Old person/part of growing old	Lay concept	Dementia is a normal ageing process	Body	Older age paradigm
“They think it’s do with old age” (Parveen et al, 2017, p. 738)^17^	Old age	Lay concept	Dementia is a normal ageing process	Body	Older age paradigm
“I think its old age because old age takes everything . . .” (Owokuhaisa et al, 2020, p. 5)^9^	Old age	Lay concept	Dementia is a normal ageing process	Body	Older age paradigm
“People with dementia was HIV-positive and epileptic . . .” (Kehoua et al, 2019, p. 171)^63^	Infections	Biomedical concept	Dementia is a disease	Body	Disease and illness paradigm
“Dying of brain cells” (Parveen et al, 2017, p. 737)^17^	Dying of brain cell	Biomedical concept	Dementia is a disease	Body	Disease and illness paradigm
“Can it be inherited?” (Parveen et al, 2017, p. 737)^17^	Gene	Biomedical concept	Dementia is a disease	Body	Disease and illness paradigm
“. . . she is having such sickness” (Nwakasi et al, 2021, p. 1453)^11^	Sickness	Identity theory	Dementia is a sickness	Body	Dementia identity paradigm
“. . . Had it not been that he is old we will say he is mad” (Agyeman et al, 2019, p. 909)^12^	He is mad	Identity theory	Dementia is madness	Mind	Dementia identity paradigm
“They [the villagers] called me asking and saying if it is madness that is happening to my gradma” (Nwakasi et al, 2021, p. 1453)^11^	Madness	Identity theory	Dementia is madness	Mind	Dementia identity paradigm

## Codes and Themes

### Strengths and Limitations

The study provided a greater description of methods and higher-order interpretation of the dementia phenomenon, illuminating a lay conceptual understanding of dementia among Black African populations. The reviewer used the evidence synthesis methods to transcend the individual studies’ findings developed by the authors of the primary qualitative studies to create higher-order themes. However, the methods were subject to selection biases, and the findings underrepresented the Black African populations as the reviewer developed analytical rather than individual descriptive analyses of the culture. Synthesizing qualitative data across studies from diverse African cultural contexts can be challenging as culture is a complex whole rather than just an analytical simplification.

## Results

### A: The Main Study Findings

### B: Themes (Findings From the Current Review)

The reviewer developed higher-order themes. Four themes emerged from the current research constituting dementia.

**Table 8.** Summary of Each Main Study’s Findings.

	Main studies	Findings
1	Adamson (2001)	Awareness of dementia and an understanding of its causes were limited. However, most participants were aware of the condition “dementia” but used different terms to describe the disorder.
2	Adamson and Donovan (2005)	The experience of an informal carer had many similarities to the experience of chronic illness, and participants described highly disruptive elements to change in relationships.
3	Adebiyi et al (2016)	The findings revealed the presence of enacted stigma in the community, suggesting a need for stigma-reducing interventions.
4	Agyeman et al (2019)	The findings revealed that symptoms of cognitive impairment were generally linked to witchcraft and inexorable bodily decline understood to be characteristic of “normal” ageing.
5	Armstrong et al (2022)	The study identified four main themes, and the findings suggested that participants’ relationships with their community, knowledge of dementia services, identity and faith influenced their experience of dementia.
6	Baghirathan et al (2020)	The study provided two categories. Dementia was viewed as part of a broader process of cultural socialization in which the norms, attitudes, and practices that act as a community’s unique identifiers are passed across generations. Dementia stigma created a barrier to seeking assistance outside the family system.
7	Berwald et al (2016)	Dementia was viewed as a white person’s illness, and there were concerns about stigma. The study suggested that Black Africans lack dementia knowledge and rely on community support for dementia. Forgetfulness was viewed as a normal ageing process.
8	Botsford et al (2011)	Participants engaged in an ongoing process of “redefining relationships.” Participants accommodated the changes associated with dementia into their lives rather than seeking help.
9	Gurayah (2015)	Three themes emerged: the views and responsibilities of the caregiver, the impact of caregiving, and services to assist the caregiver.
10	Hindley et al (2016)	A conceptualization of dementia by the healers as a “normal ageing” process. Dementia was also conceptualized by the informal carer and people with dementia as witchcraft. Dementia was diagnosed and treated with herbs and prayers. All the participants were open and willing to collaborate with allopathic healthcare services.
11	Jacobs et al (2022)	The study shows that people living with dementia and carers experienced high levels of internalized stigma related to negative public attitudes, which were associated with high levels of isolation, health system unpreparedness and limited access to support.
12	Kakongi et al (2020)	The results show that the choice for each point of dementia care depended on several factors, including dementia knowledge and beliefs. Hospital points of care were more frequent at initial health care visits, while places of worship took the lead at subsequent visits.
13	Kehoua et al (2019).	The leaders of syncretic churches and traditional healers were the first therapeutic itineraries for people with dementia. People with dementia were socially stigmatized and accused of witchcraft.
14	Lawrence et al (2008)	Carers were identified as holding a “traditional” or “non-traditional” caregiving ideology. Participants conceptualized caregiving as natural, expected, and virtuous—these informed feelings of fulfillment strained carers’ fear and attitudes toward formal services.
15	Mahomed and Pretorius (2022)	Thematic analysis of the data generated four significant themes. One theme included Awareness, Knowledge, and Education.
16	Mahomed and Pretorius (2021)	Findings suggested a shift in perception away from the cultural paradigm.
17	Mkhonto and Hanssen (2018)	Two main themes emerged: People with dementia were perceived as witches, and the study identified the need for dementia knowledge and education.
18	Mukadam et al (2011)	BME ethnic carers tended to delay help-seeking until they could no longer cope, and dementia symptoms were perceived as a normal part of ageing. Beliefs affected their level of engagement with formal services.
19	Mushi et al (2014)	Four themes emerged: low knowledge of dementia, conceptualization of dementia as a normal ageing process, and belief that dementia is a curse or witchcraft. PWDs and their carers demonstrated pluralistic behavior in seeking help from modern care, prayers, and traditional healers.
20	Nwakasi et al (2021)	The findings suggested that knowledge deficit, poor awareness, and traditional spiritual beliefs drove dementia-related stigmatization in Nigeria.
21	Owokuhaisa et al (2020)	Five themes emerged: Labeling the illness, presentation of the person with dementia, causation of dementia, impact of the disease, and how to address unmet needs in dementia care. Dementia was commonly referred to as “mental disorientation,” and dementia was also perceived as a normal part of ageing. The causes of dementia were attributed to witchcraft, life stress, infections, and poor nutrition.
22	Parveen et al (2017)	All groups attributed a biological cause to dementia. African and Caribbean groups focused on religion and spirituality as a method for personal control or cure. Dementia was associated with stigma. African populations rely on community support and are underrepresented in dementia specialist services.

**Table 9.** Themes (Higher-Order Themes).

Themes	Constituting dementia
Dementia witchcraft paradigm	Witchcraft, sorcery, curses, evil eye
Dementia older age paradigm	Normal ageing process, ageing, and older age
Dementia disease and illness paradigm	Illnesses, infections, genetics, injuries, forgetfulness, memory loss, poverty, and environmental factors
Dementia identity paradigm	Sickness, madness, craziness, insanity, ethnicity, religiosity, and spirituality

#### Dementia Witchcraft Paradigm

The findings highlighted the dementia witchcraft paradigm theme, which illuminated how dementia was constituted as sorcery, curses, and evil eye but broadly as witchcraft among the Black African populations, as demonstrated in the excerpts. For example,

You know in the village; they will think that somebody is bewitching [or casting evil spell on] that person [with dementia] . . .^10^ (p. 1452)

Indeed, dementia perceived as witchcraft was also linked to spirituality and religiosity:

I have already heard of dementia . . . but there are demons in all dementia: Matthew 17 verse 21: This type of demon comes out only through prayer and fasting.^63^ (p. 170)

They use to give sacrifice to the ancestors so due to her age she cannot afford to give sacrifice to them so they get annoyed and cause all these problems.^11^ (p. 133)

#### Dementia Older Age Paradigm

The findings illuminated the dementia older age paradigm theme, which highlights how dementia was constituted as a normal ageing process, ageing, and older age as showcased in the excepts. For example,

Where I come from there’s no definition of dementia, an old person is starting to forget, it’s part of growing old.^16^ (p. 5)

I know age is one [cause] of dementia, and I believe we all know that the older we become, the more our mind becomes stale.^62^ (p. 271)

#### Dementia Disease and Illness Paradigm

The results also illuminated the dementia disease and illness theme, widely drawn as infections, genetics, injuries, forgetfulness, memory loss, poverty, and environmental conditions, as can be seen in the showcased excerpts. For example,

People with dementia were HIV-positive and epileptic . . .^63^ (p. 171)

The majority get the disease of forgetfulness . . .^9^ (p. 6)

Moreover, the findings illuminated dementia as an organic pathology:

Dying of brain cells’^17^ (p. 737)

Dementia was also narrated as an illness:

Then, when I was allowed, I was told, “he is ill” . . .^51^ (p. 9)

#### Dementia Identity Paradigm

Finally, the findings highlighted the dementia identity paradigm theme, illuminating that dementia was constituted through a process of differences defined in social relationships and behavior, such as sickness, madness, craziness, insanity, ethnicity, disability, religiosity, and spirituality, as showcased in the excerpts. For example,

Had it not been that he is old we will say he is mad^12^ (p. 909)

. . . she is having such sickness^10^ (p. 1453)

Ethnicity/race resonated strongly in the studies:

When you talk about dementia . . . this is a White, old White people’s disease, it’s not seen as Black people have dementia^16^ (p. 7)

## Comparison of the Findings in Africa and the UK

The evidence synthesis shows that the findings from the UK and Africa differ and are similar. The UK findings lack authenticity for Black Africans and, therefore, are invalid for Black African populations compared to the findings of the studies in Africa. However, the lay knowledge of dementia as older age and ageing, as well as dementia as a disease and illness, were reciprocally translated in both the UK and studies in Africa with similar logic and meanings that explained the dementia phenomenon among the Black African populations, and intersected with biomedical knowledge and understanding about dementia.

However, there were inconsistencies in the dementia witchcraft paradigm as the findings in Africa refute the UK studies due to the modeling of Black African and Caribbean people in the UK studies, and the research provided no separate findings for the Black African populations in the UK. Black Africans and the Caribbean people are distinct from each other, suggesting a need for further empirical study in the UK. Nonetheless, the dementia identity paradigm theme is an emerging concept in both studies, related to social relationships that lead to dementia stigma in both Africa and the UK. The dementia identity paradigm provides a line of argument synthesis, considering dementia a social identity and a new interpretation of the dementia phenomenon among Black Africans.

Consequentially, dementia perceived as witchcraft influenced help-seeking for dementia in Africa, and in any case, dementia perceived as witchcraft in the UK by Black Africans would equally impact help-seeking for dementia. It is more likely that Black Africans will resort to seeking help for dementia in churches rather than dementia specialist services, and this can lead to stigma and abuse. So, the reviewer argues for tailored educational and training programs aimed at reducing the stigma associated with dementia and improving outreach efforts to better support the Black African communities in dealing with dementia-related issues and preventing the abuse of people with dementia.

## Discussion

Dementia is a public health burden in the UK.^20^ The high prevalence of dementia among Black Africans, coupled with their lower engagement with dementia specialist services in the UK,^16,17^ is a public health concern. The findings in this study showcased how dementia was constituted and constructed among the Black African populations. Generally, lay people’s knowledge and beliefs about dementia are not limited to biomedical constructs. People can draw on their own meaning and interpretation of their experiences of dementia in everyday life.^29,30^ Different populations can draw on dementia as defined by representations^79-81^ and can also be based on their own beliefs and culture.^5^ The evidence synthesis shows that the lay knowledge and beliefs about dementia among Black African populations were foregrounded in sociocultural distinctiveness, constituting mainly as witchcraft (eg, studies^7,9,10^), older age (eg, studies^11,12^), disease and illness (eg, studies^16,17,63^), and with social identity based on relationships (eg, studies^12,13^).

Belief in witches’ crafts is widespread in Africa.^6^ They are part of a complex body of inherited folklore,^4^ and dementia identity is constituted through differences in social identity based on relationships with others in the Black African community.^82^ In addition, dementia identity theory is situated with the mind/brain and body constructions.^83^ Hence, the identity theory of the mind/brain and body holds that the processes of the mind are identical to the state of the brain and the physical body.^83-85^ So, dementia was associated with “madness” and “craziness” in Black African communities.^12^

Being mad/crazy or witches’ crafts can be viewed as a more comprehensive cultural socialization and unique dementia constructions/constructs or identifiers,^86^ passed across the Black African community members and shaped their dementia help-seeking attitudes and behavior in the UK^19^ and across Sub-Saharan Africa.^63^ Most studies reported pluralistic behaviors (eg, study^13^) in seeking help due to labeling and stigma.^87,88^

All the studies in Africa with Black African populations associated dementia with witchcraft. However, the association with witchcraft is inconsistent with the wider UK literature on Black African populations in the UK as reviewed. The studies on BAME in the UK suggested that Black Africans in the UK lacked knowledge of dementia.^16^ It further suggested that Black African populations in the UK rely on community, friends, and families for dementia support and advice. Black African populations are underrepresented in UK dementia specialist services and use churches for dementia support.^17^ Indeed, religion and spirituality are integral parts of Black African culture, and it is more likely for Black African populations in the UK to fall back on those services with which they are more familiar.^19^

Nevertheless, the suggestion that Black Africans in the UK lacked dementia knowledge^16^ is unreliable as the studies had no separate data for the Black African population. Thus, the conclusion drawn from the study is not credible, invalid, and lacks authenticity for the Black African community in the UK. However, we expect dementia knowledge among the Black African populations in the UK to vary based on demographics such as educational level, occupation, age, gender, and immigration status. Most importantly, dementia knowledge may have evolved in the Black African community in the UK.

Therefore, to address these gaps, this study recommends further empirical research to explore the lay knowledge toward dementia among Black African populations in the UK. The study must focus on methodological adaptations in order to continually make the research culturally sensitive.^89^ Incorporating the views, experiences, perspectives, and practices of the Black African populations in the UK in all stages of the research process is essential because studies that ignore or do not account for the cultural perspectives of participants are invalid.^89^ Indeed, future studies must be informed by a grounded theory approach. More importantly, longitudinal studies among Black African populations are needed to track changes in dementia perceptions over time.

## Conclusion

The evidence synthesis shows that dementia is culturally embedded among the Black African populations and associated with stigma and abuse. However, the UK studies do not wholly inform us about the lay knowledge and beliefs about dementia among Black African populations in the UK. Previous studies in the UK lack authenticity for Black African populations and, therefore, are invalid. Moreover, most of the UK studies are outdated. There is an essential need for future studies among the Black African populations in the UK and a need for a longitudinal study to track changes in dementia perceptions in the group over time.

### Recommendations

There is a need for culturally tailored educational and training programs aimed at reducing the stigma associated with dementia and improving community outreach efforts to better support Black African populations in dealing with dementia-related issues. Thus, this study recommends strong partnership or collaboration between medical doctors, community leaders, and applied medical anthropologists in dementia practice to help resolve the dementia stigma and prevent the abuse of people with dementia in Black African communities.

### Implications for Policy and Practice

The study has broad implications for dementia policy and practice. A better understanding of the lay knowledge and beliefs about dementia held by Black Africans can improve dementia help-seeking and care, help prevent the stigma and abuse associated with certain beliefs about dementia, and provide culturally sensitive and tailored support for Black African communities. This study contributes to the evidence base for dementia practice in the Black African communities in the UK and Africa.
